# Supplementary material for: Antibiotic-induced microbiome depletion alters metabolic homeostasis by affecting gut signaling and colonic metabolism
Source: Nat Commun. 2018 Jul 20;9:2872. doi: 10.1038/s41467-018-05336-9 (PMC6054678; doi:10.1038/s41467-018-05336-9)
Supplement: Supplementary file 1 — Supplementary Information [file 41467_2018_5336_MOESM1_ESM.pdf]

**Supplementary Materials**

Supplementary Figures 1-6

Supplementary Tables 1 & 2

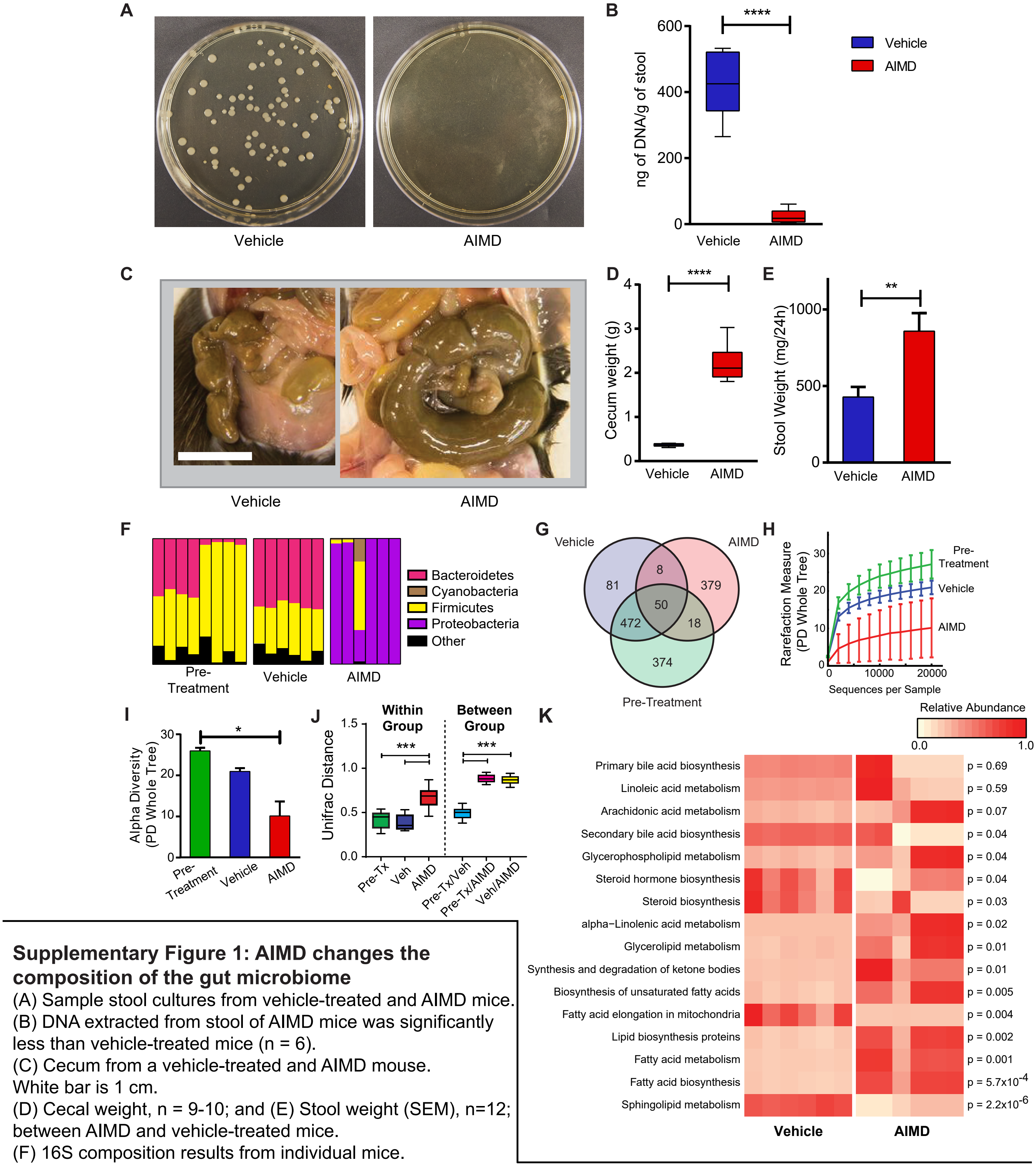


**Supplementary Figure 1: AIMD changes the composition of the gut microbiome**

(A) Sample stool cultures from vehicle-treated and AIMD mice.
(B) DNA extracted from stool of AIMD mice was significantly less than vehicle-treated mice (n = 6).
(C) Cecum from a vehicle-treated and AIMD mouse. White bar is 1 cm.
(D) Cecal weight, n = 9-10; and (E) Stool weight (SEM), n=12; between AIMD and vehicle-treated mice.
(F) 16S composition results from individual mice.
(G) Number of shared OTUs between conditions. Rarefaction plot (H) and PD-whole tree plot (I) show that α-diversity (SEM) was decreased in AIMD mice.
(J) Unifrac distances showing β-diversity differences. The pre-treatment and vehicle treatment microbiomes were similar to each other based on UniFrac distance, whereas the AIMD microbiome had high UniFrac distances both within its own group and with the other conditions.
(K) PICRUSt results of lipid metabolism pathways in the gut microbiome of AIMD and Vehicle mice.
For boxplots, center is mean, box is 25th to 75th percentile, whiskers are 5th to 95th percentile.
Mann-Whitney U test, except for (I) which was a Kruskal-Wallis test; * $p<0.05$, ** $p<0.01$, *** $p<0.001$.

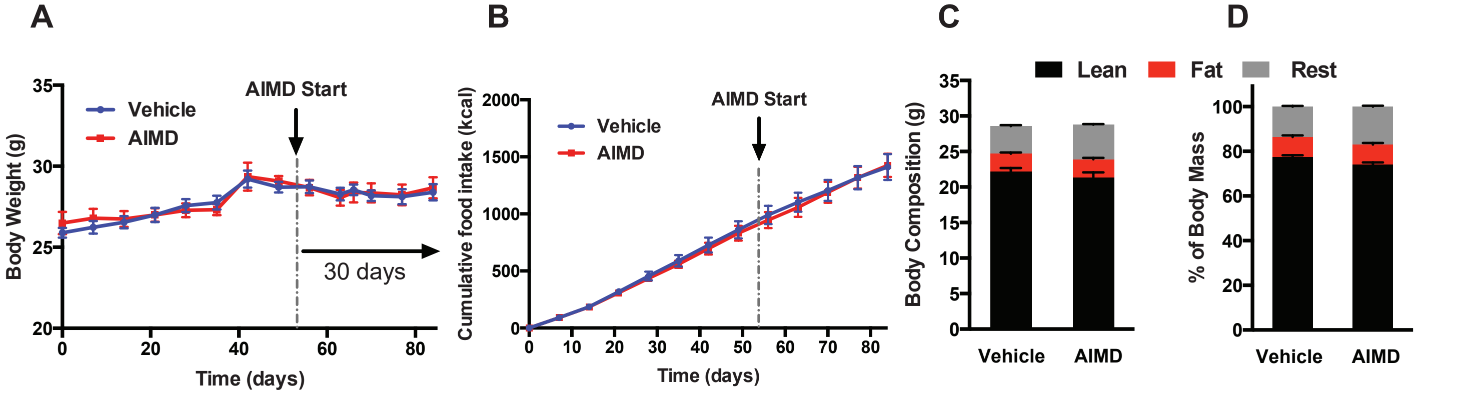


**Supplementary Figure 2. Long-term twice daily AIMD protocol does not affect body weight, food consumption, and body composition of the mice.**

Mice received the antibiotics cocktail twice a day for 30 days.

Body weight (A), food consumption (B) and body composition in grams (C) or as a percentage of total body weight (D) are not different between control and treated mice. All error bars are SEM.

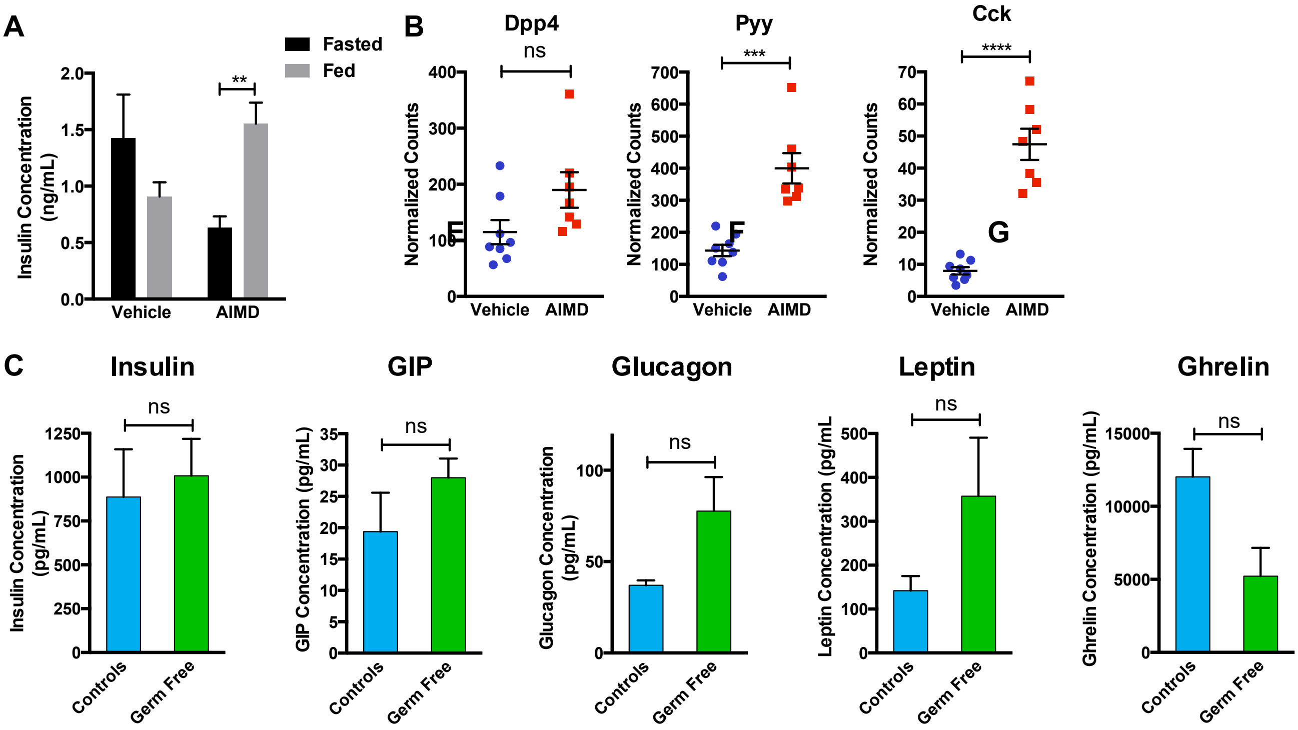


## Supplementary Figure 3: Microbiome depletion in AIMD or germ-free mice leads to increased level of GLP1 and other gut hormones.

(A) Serum level of insulin after 16 hours of fasting (asted) or 15 min after an oral bolus of glucose (1g/kg BW; Fed). Blood was collected on Ddp-4 inhibitor coated tubes (n=5/group).

(B) Quantification by RNA sequencing of Dpp4, Pyy and Cck mRNA expression in the cecum (n=7-8/group). Adjusted p-value, * $p<0.05$, ** $p<0.01$, *** $p<0.001$, **** $p<0.0001$.

(C-G): Serum endocrine panel in germ-free mice - serum levels of total insulin (C) , GIP (D), Glucagon (E), Leptin (F), Ghrelin (G) (n=5/group). Student's t-test, * $p<0.05$, ** $p<0.01$, *** $p<0.001$. All error bars are SEM.

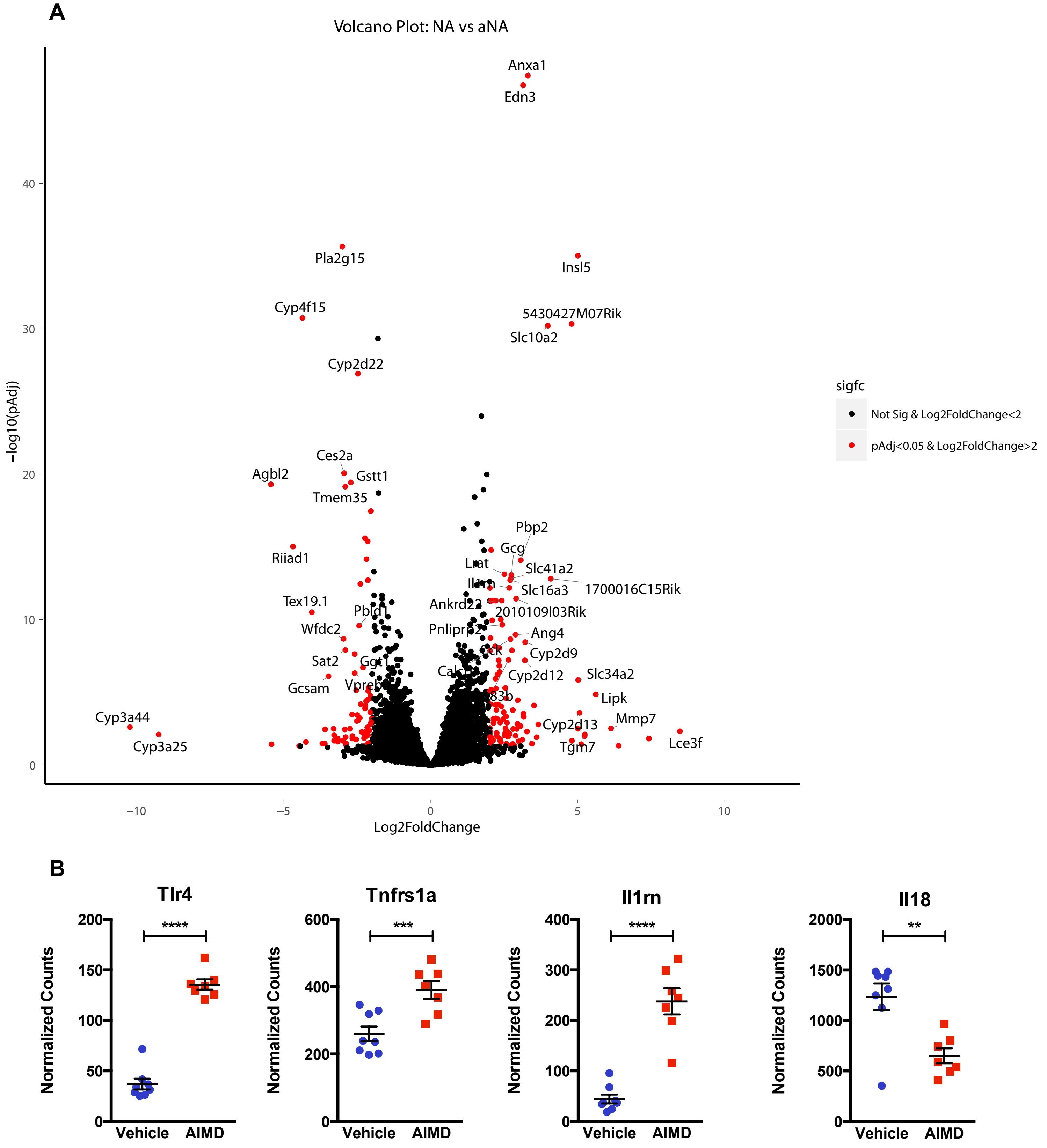


**Supplementary Figure 4: AIMD induces major changes in the cecal transcriptome, including the genes expressing inflammatory cytokines.**

(A) Volcano plot of RNA-sequencing data.

(B) Expression level (and SEM) of selected pro and anti-inflammatory cytokines in the cecum.

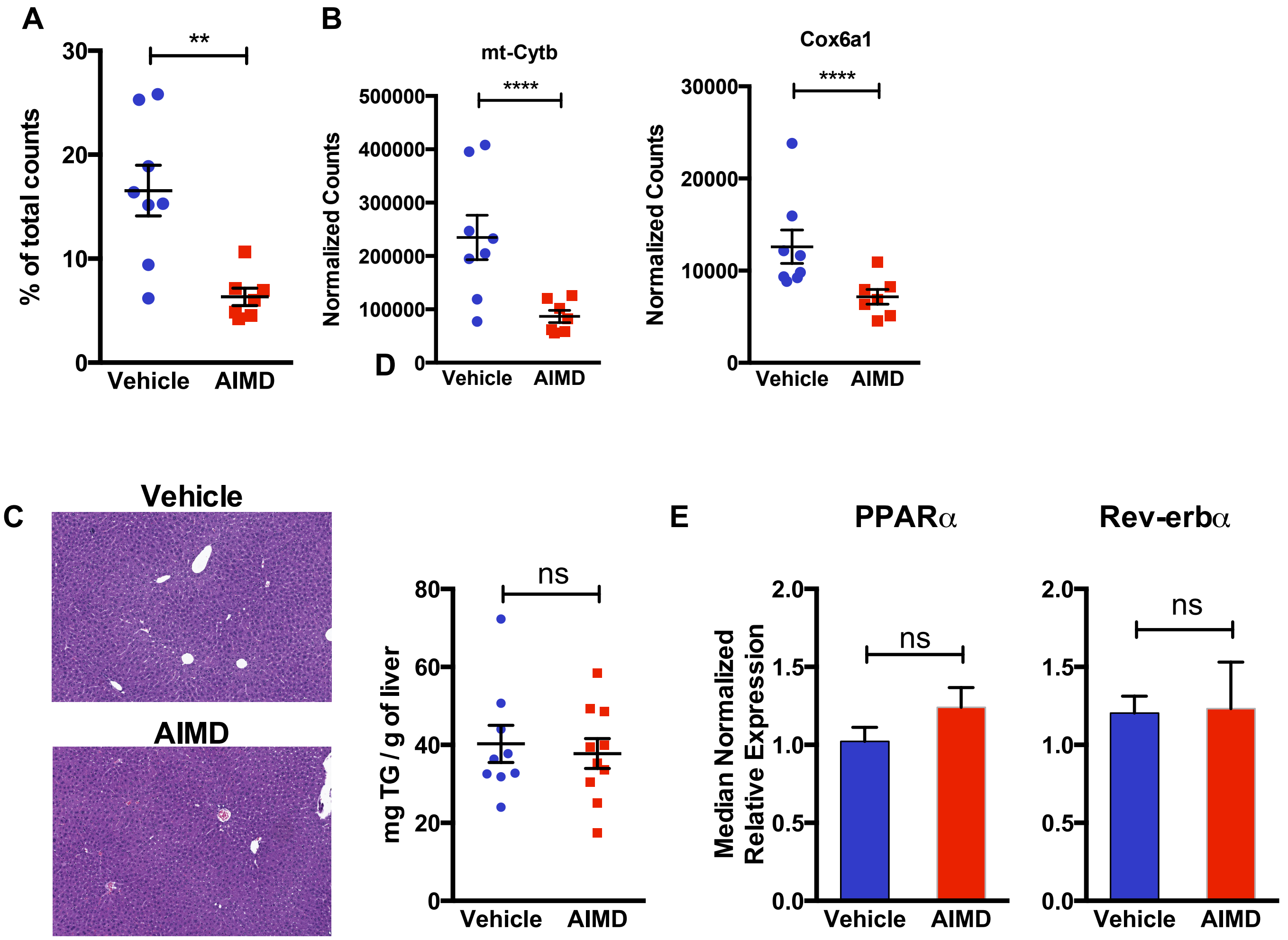


**Supplementary Figure 5: Mitochondria are reduced in the cecum of AIMD mice and hepatic lipid metabolism is unaffected by AIMD treatment.**

(A) Percentage of total cecal RNA-sequencing reads mapping to mitochondrial chromosome M.
(B) Quantification by RNA sequencing of mt-Cytband Cox6a1 mRNA levels in the cecum (n = 7-8/group).
Adjusted p-value, * p<0.05, ** p<0.01, *** p<0.001, **** p<0.0001.
(C) Representative H&E staining of the liver of vehicle and AIMD mice.
(D) Hepatic triglyceride levels (n=9-10/group).
(E) Quantification by RT-qPCR of PPARα and Rev-erbα mRNA levels in the liver (n=8/group).
All error bars are SEM.

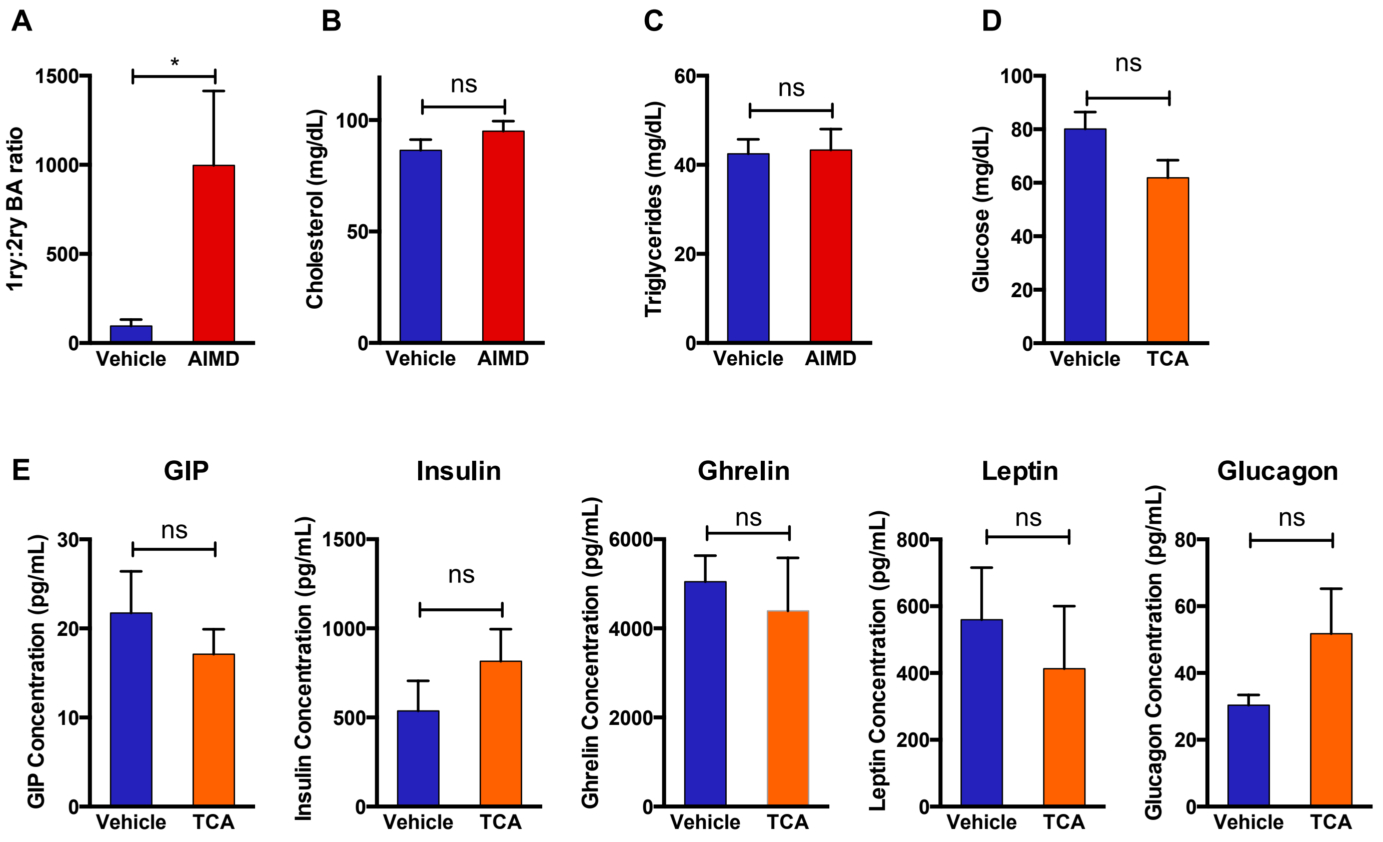


**Supplementary Figure 6: Serum cholesterol and TG are unchanged in AIMD mice; TCA gavage affects serum endocrine profile.**

(A) Ratio of primary to secondary bile acids in the serum.
(B) Serum cholesterol level in AIMD mice (n=8-10/group).
(C) Serum triglycerides level in AIMD mice (n=8-10/group).
(D) Serum glucose levels in TCA mice (n=4/group).
(E) Serum endocrine panel in TCA mice - serum levels of GIP, Ghrelin, Leptin and Glucagon.
Unpaired t-test, * p<0.05, ** p<0.01, *** p<0.001. All error bars are SEM.

## SUPPLEMENTARY EXPERIMENTAL PROCEDURES

Supplementary Table 1. Measurements of common fecal bile acids (in nmol/mg)

| | Vehicle-treated n=11 | AIMD n=12 | p-value |
|---|---|---|---|
| **Primary Bile Acids** | | | |
| Cholic Acid (CA) | 2.04 *(0.993)* | 0.02 *(0.003)* | 0.008 |
| α-Muricholic Acid (aMCA) | 10.22 *(3.989)* | 0.00 *(0.000)* | 1.40E-05 |
| β-Muricholic Acid (bMCA) | 41.73 *(15.660)* | 0.08 *(0.028)* | 1.00E-06 |
| Taurocholic Acid (TCA) | 0.86 *(0.379)* | 6.38 *(1.708)* | 4.40E-05 |
| Tauro-α-Muricholic Acid (TaMCA) | 5.22 *(1.113)* | 0.93 *(0.324)* | 1.43E-04 |
| Tauro-β-Muricholic Acid (TbMCA) | 6.53 *(1.611)* | 23.59 *(7.582)* | 0.002 |
| Taurochenodeoxycholic Acid (TCDCA) | 0.16 *(0.039)* | 0.37 *(0.042)* | 0.001 |
| **Total Primary Bile Acids** | 66.76 *(22.190)* | 31.37 *(8.405)* | 0.027 |
| **Secondary Bile Acids** | | | |
| Deoxycholic Acid (DCA) | 5.89 *(1.794)* | 0.32 *(0.080)* | 1.32E-04 |
| Hyocholic Acid (HCA) | 2.16 *(0.616)* | 0.00 *(0.002)* | 0.003 |
| ω-Muricholic Acid (wMCA) | 44.83 *(44.830)* | 0.00 *(0.004)* | 1.00E-06 |
| Taurodeoxycholic Acid (TDCA) | 0.91 *(0.282)* | 0.00 *(0.000)* | 1.40E-05 |
| Tauroursodeoxycholic Acid (TUDCA) | 0.34 *(0.035)* | 0.34 *(0.089)* | 0.316 |
| Taurolithocholic Acid (TLCA) | 0.14 *(0.029)* | 0.01 *(0.005)* | 3.00E-06 |
| Taurohyodeoxycholic Acid (THDCA) | 0.38 *(0.051)* | 0.34 *(0.089)* | 0.134 |
| **Total Secondary Bile Acids** | 54.66 *(14.850)* | 1.03 *(0.164)* | 1.00E-06 |

Note: Mean (SEM); p-value calculated with Mann-Whitney U test

Supplementary Table 2: Measurements of common serum bile acids (in μM)

| | Vehicle-treated n = 10 | AIMD n = 9 | p-value |
|---|---|---|---|
| **Primary Bile Acids** | | | |
| Cholic Acid (CA) | 0.08 *(0.049)* | 9.59E-05 *(2.97E-05)* | 2.20E-05 |
| α-Muricholic Acid (aMCA) | 1.05E-04 *(7.10E-05)* | 3.77E-05 *(3.77E-05)* | 0.5975 |
| β-Muricholic Acid (bMCA) | 0.03 *(0.017)* | 3.83E-03 *(2.24E-03)* | 0.0133 |
| Taurocholic Acid (TCA) | 0.32 *(0.211)* | 0.55 *(0.337)* | 0.0101 |
| Tauro-α-Muricholic Acid (TaMCA) | 13.08 *(7.724)* | 126.50 *(81.450)* | 0.4470 |
| Tauro-β-Muricholic Acid (TbMCA) | 42.81 *(28.440)* | 130.10 *(80.620)* | 0.0101 |
| Taurochenodeoxycholic Acid (TCDCA) | 0.02 *(0.011)* | 0.06 *(0.037)* | 0.0101 |
| **Total Primary Bile Acids** | 56.32 *(36.440)* | 257.30 *(162.400)* | 0.0101 |
| **Secondary Bile Acids** | | | |
| Deoxycholic Acid (DCA) | 0.05 *(0.012)* | 0.02 *(0.002)* | 0.0015 |
| Hyocholic Acid (HCA) | 0.00 *(0.000)* | 1.18E-04 *(8.29E-05)* | 0.2105 |
| ω-Muricholic Acid (wMCA) | 0.03 *(0.010)* | 0.00 *(0.000)* | 2.20E-05 |
| Taurodeoxycholic Acid (TDCA) | 0.12 *(0.077)* | 0.00 *(0.000)* | 2.20E-05 |
| Tauroursodeoxycholic Acid (TUDCA) | 0.03 *(0.017)* | 0.08 *(0.047)* | 0.0101 |
| Taurolithocholic Acid (TLCA) | 7.10E-04 *(4.12E-04)* | 6.40E-05 *(2.49E-05)* | 0.0041 |
| Taurohyodeoxycholic Acid (THDCA) | 0.02 *(0.010)* | 3.78E-06 *(3.78E-06)* | 2.20E-05 |
| **Total Secondary Bile Acids** | 0.24 *(0.125)* | 0.10 *(0.047)* | 0.0435 |

Note: Mean (SEM); p-value calculated with Mann-Whitney U test.
