# Supplementary material for: Antibiotic-induced microbiome depletion alters metabolic homeostasis by affecting gut signaling and colonic metabolism
Source: Nat Commun. 2018 Jul 20;9:2872. doi: 10.1038/s41467-018-05336-9 (PMC6054678; doi:10.1038/s41467-018-05336-9)
Supplement: Supplementary file 3 — Description of Additional Supplementary Files [file 41467_2018_5336_MOESM3_ESM.docx]

**Description of Additional Supplementary Files**

**File Name: Supplementary Data 1**

**Description:**

Supplementary Data 1 contains the 16S sequencing annotations: This EXCEL file includes 5 pages with each for a part of the phylogenic tree (phylum, class, order, family, and genus). Column A has the OTUs assigned to the phylogeny, Column B-I has the 8 samples from the pre-treatment condition (Pretx 1-8), Column J-O has the 6 samples from the vehicle-treated condition (NA 1-6), and Column P-U has the 6 samples from the antibiotic-treated conditions (aNA 1-6). The numbers in the cells represent the percent reads.

**File Name: Supplementary Data 2**

**Description:**

Supplementary Data 2 contains the results of PICRUSt analysis: This EXCEL file includes 3 pages with each a different level of the KEGG Orthology Pathway (Level 1-3). Column A has the KEGG Orthology Pathway abundances, Column B-I has the 8 samples from the pre-treatment condition (Pretx 1-8), Column J-O has the 6 samples from the vehicle-treated condition (NA 1-6), and Column P-U has the 6 samples from the antibiotic-treated conditions (aNA 1-6). The numbers in the cells represent the number of hits in that pathway from 16S sequencing results.

**File Name: Supplementary Data 3**

**Description:**

Supplementary Data 3 contains the annotated RNA-seq results. This EXCEL file includes one page. Column A contains the gene. Column B contains the mean normalized count of vehicle-treated samples. Column C includes the log2 fold change of antibiotic-treated condition, Column D has the raw p-value of the difference using a negative binomial Wald test, and Column E has the adjusted p-value which is corrected for multiple hypothesis testing with the Benjamini-Hochberg method.
